# Leptin and the Regulation of Renal Sodium Handling and Renal Na^+^-Transporting ATPases: Role in the Pathogenesis of Arterial Hypertension

**DOI:** 10.2174/157340310790231644

**Published:** 2010-02

**Authors:** Jerzy Bełtowski

**Affiliations:** Dept. of Pathophysiology, Medical University, Lublin, Poland

**Keywords:** leptin, renal Na^+^ transport, Na^+^, K^+^-ATPase, obesity, arterial hypertension, oxidative stress.

## Abstract

Leptin, an adipose tissue hormone which regulates food intake, is also involved in the pathogenesis of arterial hypertension. Plasma leptin concentration is increased in obese individuals. Chronic leptin administration or transgenic overexpression increases blood pressure in experimental animals, and some studies indicate that plasma leptin is elevated in hypertensive subjects independently of body weight. Leptin has a dose- and time-dependent effect on urinary sodium excretion. High doses of leptin increase Na^+^ excretion in the short run; partially by decreasing renal Na^+^,K^+^-ATPase (sodium pump) activity. This effect is mediated by phosphatidylinositol 3-kinase (PI3K) and is impaired in animals with dietary-induced obesity. In contrast to acute, chronic elevation of plasma leptin to the level observed in patients with the metabolic syndrome impairs renal Na^+^ excretion, which is associated with the increase in renal Na^+^,K^+^-ATPase activity. This effect results from oxidative stress-induced deficiency of nitric oxide and/or transactivation of epidermal growth factor receptor and subsequent stimulation of extracellular signal-regulated kinases. Ameliorating “renal leptin resistance” or reducing leptin level and/or leptin signaling in states of chronic hyperleptinemia may be a novel strategy for the treatment of arterial hypertension associated with the metabolic syndrome.

## LEPTIN AND ITS ROLE IN ARTERIAL HYPERTENSION

Overweight and obesity are the leading causes of arterial hypertension worldwide. It is estimated that in highly developed countries about 2/3 cases of essential hypertension can be attributed to excess body weight. Obesity becomes also an increasing contributor to hypertension in developing countries as well as in children and adolescents [1]. The pathogenesis of obesity-associated hypertension is complex and incompletely understood, however, as in other forms of hypertension; abnormal renal sodium handling and disturbed regulation of the vascular tone play a predominant role [2]. Recent studies indicate that adipose tissue is not only a site of triglyceride storage but also an active endocrine organ which secretes multiple biologically active mediators called adipokines, such as leptin, adiponectin, resistin, visfatin, apelin, etc.; production of which is either up-regulated or downregulated in obese subjects [3]. At least some of these adipokines exert physiological effects on the cardiovascular system, and abnormalities of their production may contribute to the pathogenesis of obesity-associated complications. In this article I focus on the role of leptin – the first identified and the best characterized adipokine – in the regulation of renal Na^+^ handling. I characterize physiological effects of leptin as well as its involvement in obesity-associated hypertension.

Leptin was identified in 1994 by positional cloning of the *ob* gene responsible for the development of obesity in *ob/ob* mice [4]. It is produced by adipocytes in proportion to the amount of adipose tissue and acts on hypothalamus to decrease food intake and stimulate energy expenditure. Leptin binds to plasma membrane receptors existing in at least 6 isoforms, Ob-Ra through Ob-Rf. The main or “long” isoform, Ob-Rb, contains the longest intracellular domain and, upon leptin binding, stimulates Janus kinases (JAK) which phosphorylate tyrosine residues of signal transducers and activators of transcription (STAT) proteins. Phosphorylated STAT proteins translocate to the nucleus and regulate transcription of target genes. Short isoforms of the leptin receptor, Ob-Ra, Ob-Rc, Ob-Rd and Ob-Rf, have shorter intracellular domains and cannot activate JAK-STAT pathway but may perform signal transduction through other mechanisms such as phosphatidylinositol 3-kinase (PI3K), mitogen-activated protein kinases (MAPK), etc. Ob-Re is a soluble form of the leptin receptor which circulates in the blood and is a major leptin-binding protein. Apart from hypothalamus, leptin receptors are contained in many peripheral tissues including the cardiovascular system [5].

Homozygous *ob/ob* mice are extremely obese due to inherited lack of leptin. In contrast, animals with dietary-induced obesity as well as most obese humans are characterized by hyperleptinemia. Two other leptin-related animal models of obesity exist: *db/db* mice which lack Ob-Rb but have functional other receptor subtypes, and obese Zucker *fa/fa* rats bearing mutation within the extracellular domain of the leptin receptor which reduces the affinity for leptin of all receptor isoforms. Both these animal strains are severely obese, hyperleptinemic, leptin-resistant and insulin-resistant.

Several lines of evidence suggest the role of leptin in the pathogenesis of arterial hypertension: (1) chronic leptin administration [6] or transgenic overexpression [7] increases blood pressure (BP) in experimental animals, (2) BP is not increased in leptin-deficient ob/ob mice despite severe obesity, and supplementation of leptin to these animals increases BP despite reducing body weight [8], (3) some studies indicate that plasma leptin concentration is higher in patients with arterial hypertension independently of body weight [9]. These data raise interest about possible effects of leptin in the cardiovascular system.

## ABNORMALITIES OF RENAL Na^+^ HANDLING AND RENAL Na^+^,K^+^-ATPASE IN THE PATHOGENESIS OF ARTERIAL HYPERTENSION

Apart from vascular tone, renal sodium handling is the second major factor regulating blood pressure. Although determined by many factors including renal perfusion, glomerular filtration, etc., sodium excretion is regulated mainly at the level of renal tubules. More than 99% of filtered Na^+^ is normally reabsorbed throughout the nephron and thus even very small changes in the rate of reabsorption may cause marked alterations of Na^+^ excretion leading ultimately to disturbances of Na^+^ balance, extracellular fluid volume and blood pressure [10]. Sodium reabsorption is a two-step process: first Na^+^ ions passively enter the tubular cells through transporters localized in the apical plasma membrane and then are actively extruded from the cell to the peritubular space against the concentration gradient. Whereas passive transporters change along the nephron (sodium/proton exchanger, furosemide-sensitive sodium/-potassium/chloride symporter, sodium/chloride symporter and epithelial sodium channel are predominant apical transporters in the proximal convoluted tubule, medullary thick ascending limb, distal convoluted tubule and collecting duct, respectively), active step of Na^+^ reabsorption is driven mainly by Na^+^,K^+^-ATPase in all nephron segments. Both steps of Na^+^ transport are subjects to complex neurohormonal regulation. Indeed, most, if not all, hormones, mediators and neurotransmitters involved in the regulation of Na^+^ balance affect apical passive transporters and/or basolateral Na^+^,K^+^-ATPase in specific nephron segments. Those factors which stimulate Na^+^ transport (e.g. angiotensin II, catecholamines, mineralocorticoids, glucocorticoids) ultimately reduce sodium excretion and increase blood pressure, whereas other mediators such as dopamine, cardiac natriuretic peptides, nitric oxide, etc. have the opposite effect [11].

Abnormalities of the regulation of Na^+^ transport play a pivotal role in the pathogenesis of arterial hypertension. For example, Na^+^,K^+^-ATPase activity in the proximal tubule is higher in spontaneously hypertensive rat (SHR) than in normotensive Wistar-Kyoto rat even before the development of hypertension [12-14]. In addition, abundance of Na^+^,K^+^-ATPase γ-subunit protein is greater in the proximal tubule of SHR [15]. Interestingly, in hypertensive rats a greater fraction of total cellular Na^+^,K^+^-ATPase α_1_ subunit is contained in the basolateral membrane resulting in the enhancement of Na^+^ transport [16]. In Dahl salt-sensitive rat, a glutamine for leucine substitution within the Na^+^,K^+^-ATPase α_1_ subunit gene is associated with enhanced Na^+^ reabsorption [17-19]. In addition, inhibitory effect of dopamine on tubular sodium pump is impaired in both these rat models of genetic hypertension [20, 21]. Mutations within the gene encoding cytoskeletal protein adducin, which result in the enhanced Na^+^ reabsorption, are observed in many hypertensive humans as well as in Milan hypertensive rat strain [22, 23]. These mutations increase the activity of Na^+^,K^+^-ATPase by impairing its endocytosis from the basolateral plasma membrane to inactive intracellular pool [24, 25]. Several forms of monogenic hypertension in humans are also associated with the mutations of genes which encode sodium transporters or components of their regulatory pathways (e.g. gain-of-function mutations of epithelial Na^+^ channel in the Liddle syndrome or mutations of WNK kinases which increase the activity of thiazide-sensitive Na^+^/Cl^-^ cotransporter in the Gordon’s syndrome). These data indicate that enhanced Na^+^ reabsorption, in some cases induced by overactivation of sodium pump, plays a major role in the development of hypertension.

Abnormalities of sodium transport regulation have also been implicated in the pathogenesis of hypertension associated with the metabolic syndrome. For example, the ability of dopamine to inhibit Na^+^,K^+^-ATPase-driven Na^+^ reabsorption by tubular cells is impaired, whereas the stimulatory effect of angiotensin II is enhanced in obese insulin resistant Zucker *fa/fa* rats [26, 27]. Strazzullo *et al*. [28] have demonstrated that Na^+^ reabsorption in the proximal tubule is enhanced in men with abdominal obesity. It has also been shown that renal effect of atrial natriuretic peptide is impaired in obese subjects [29]. Taken together, these data indicate that abnormalities of renal Na^+^ handling play an important role in the development of hypertension associated with the metabolic syndrome.

## ACUTE NATRIURETIC EFFECT OF LEPTIN

Many studies have demonstrated that although leptin stimulates sympathetic nervous system (SNS), it has no acute effect on blood pressure, which suggests that SNS activation is balanced by opposite depressor mechanisms. At least two such mechanisms have been described: stimulation of endothelial nitric oxide production and natriuresis. Serradeil-Le Gal and coworkers [30] first identified specific leptin binding sites in the renal medulla and demonstrated significant increase in diuresis following intraperitoneal leptin injection in mice. Subsequently, several studies demonstrated that leptin administered intraperitoneally [31], intravenously [32, 33], or locally into the renal artery [34] increased natriuresis without affecting renal blood flow, glomerular filtration rate and potassium excretion, suggesting that natriuretic effect of leptin results from inhibiting tubular Na^+^ reabsorption. We have demonstrated that systemically [31, 35] or locally [36] administered leptin induces a time- and dose-dependent decrease in Na^+^,K^+^-ATPase activity in the renal medulla. Specific leptin binding sites were identified in the renal medulla, presumably in medullary collecting duct [37]. The effect of leptin on Na^+^,K^+^-ATPase is specific since leptin did not change the activity of ouabain-sensitive H^+^,K^+^-ATPase contained in the apical membranes and involved in K^+^ reabsorption and urine acidification [35, 36].

Since both natriuretic and Na^+^,K^+^-ATPase-inhibitory effects of leptin were too rapid to be accounted for by changes in gene expression – a target of classic JAK-STAT pathway triggered by the Ob-Rb – signaling mechanisms involved in these effects were further examined [36]. Because leptin stimulates nitric oxide synthase in endothelial cells, we first studied the role of NO-cGMP system, previously demonstrated to enhance natriuresis by inhibiting renal sodium pump [38-40]. However, leptin had no effect on urinary excretion of either NO metabolites or cGMP. In addition, inhibitors of NO synthase, soluble (NO-sensitive) guanylate cyclase and protein kinase G did not abolish natriuretic effect of leptin excluding the major role of NO-cGMP pathway [36]. Moreover, leptin had no acute effect on plasma concentration of atrial natriuretic peptide (ANP) or urinary excretion of urodilatin (ANP-related peptide produced exclusively in the kidney), which stimulate membrane guanylate cyclase receptors [36]. Among other transduction pathways, inhibitors of protein kinases A or C as well as of cyclooxygenase or cytochrome P450-dependent arachidonate metabolism failed to abolish the effect of leptin. In contrast, two structurally unrelated inhibitors of phosphoinositide 3-kinase, wortmannin or LY294002, prevented leptin-induced decrease in medullary Na^+^,K^+^-ATPase activity [36]. Subsequently, we have demonstrated that wortmannin and LY2-94002 abolish also the natriuretic effect of intravenously administered leptin [41]. Although protein kinase B/Akt is a common downstream kinase of PI3K, leptin had no effect on PKB/Akt phosphorylation in the renal medulla, and specific PKB/Akt inhibitor did not abolish natriuretic effect of leptin [41]. The precise mechanism through which PI3K mediates the effect of leptin is unclear. However, PI3K has been demonstrated to mediate dopamine-induced endocytosis of Na^+^,K^+^-ATPase in renal tubular cells [42]. PI3K binds to proline-rich motif of Na^+^,K^+^-ATPase α_1_-subunit and regulates its internalization. Although this effect requires enzymatic activity of PI3K, it is not dependent on PKB/Akt [43, 44].

Physiological significance of acute natriuretic effect of leptin is incompletely understood, especially because this effect is exerted at hormone concentrations markedly exceeding the physiological range. However, Villarreal *et al*. [45] have demonstrated that blockade of endogenous leptin by a specific antibody reduced natriuresis induced in the rat by mild volume expansion. In addition, leptin expression in adipose tissue increases in response to high-salt diet [46]. These data suggest that renal effect of leptin may be relevant for maintaining Na^+^ balance, especially after ingesting a high-salt meal.

## RESISTANCE TO ACUTE NATRIURETIC EFFECT OF LEPTIN

In contrast to leptin-deficient *ob/ob* mice, plasma leptin concentration is significantly elevated in animals with dietary-induced obesity as well as in obese humans reflecting the state of hypothalamic leptin resistance. Correia *et al*. [47] proposed that obesity is accompanied by selective leptin resistance, i.e. impairment of appetite-suppressing effect and preserved sympathoexcitatory activity. This might lead, due to hyperleptinemia, to overactivation of the SNS and BP elevation. This concept has been confirmed by the experiments performed in agouti obese mice. In these animals, agouti protein – an endogenous melanocortin receptor antagonist normally expressed only in the skin - is ectopically expressed and blocks the anorectic effect of leptin-melanocortin pathway mediated by hypothalamic melanocortin receptors. Consequently, agouti mice are obese, hyperinsulinemic and leptin-resistant. In these animals either peripherally [47] or intracerebroventricularly [48] administered leptin exerts less marked effects on food intake and body weight but stimulates renal sympathetic nervous activity similarly as in lean wild-type controls. Selective leptin resistance was also observed in mice made obese by high-fat diet [49]. In addition, in the latter study chronic leptin administration increased BP to the similar extent in lean and obese groups, suggesting that renal sympathetic nervous activity is crucial for BP elevation and that acute hypertensive effect of leptin is not affected by leptin resistance.

Although initial studies suggested that resistance of hypothalamic centers to anorectic effect of circulating leptin is mainly associated with impaired hormone transport across the blood - brain barrier, later studies demonstrated that leptin resistance is also observed following central administration of this hormone due to receptor or postreceptor signaling defects. These data, together with increasing number of peripheral effects of leptin being described, led us to ask if natriuretic effect of leptin is preserved or impaired in obesity. To address this issue, we tested the effect of leptin in rats made obese by feeding highly palatable diet for 4 weeks [31]. This model of obesity is characterized by moderate increase in body weight and plasma leptin concentration but plasma glucose, lipid profile, insulin concentration and basal blood pressure are still normal. Thus, this model represents early uncomplicated phase of obesity and the results are not confounded by abnormalities of carbohydrate and lipid metabolism, insulin resistance, etc. We observed that the effects of intraperitoneally administered leptin on natriuresis and renal medullary Na^+^,K^+^-ATPase were impaired in obese animals. Thus, leptin resistance is not confined to the CNS but involves also some peripheral actions of this hormone [31].

The mechanism of leptin resistance at the renal level is not clear at present. Prolonged hyperleptinemia associated with obesity may result in the downregulation of leptin receptors or postreceptor signaling mechanisms. For example, reduced number of leptin receptors was observed in the kidney of obese rats [50] and dogs [51]. Resistance to natriuretic effect of leptin was also observed in spontaneously hypertensive rats, and renal denervation restored sensitivity to leptin in these animals [52], suggesting that increased renal sympathetic activity counteracts natriuretic effect of leptin. Among postreceptor signaling pathways, two mechanisms attracted special attention as potential contributors to leptin resistance: suppressor of cytokine signaling-3 (SOCS-3), and protein tyrosine phosphatase-1B (PTP-1B) [5]. SOCS-3 is induced upon activation of the leptin receptor and inhibits leptin signaling by binding to the receptor itself as well as to the key downstream kinase, JAK2. SOCS-3 is overexpressed in the hypothalamus in various models of leptin resistance such as dietary-induced obesity. PTP-1B dephosphorylates proteins phosphorylated after activation of the leptin receptor such as JAK2 and STAT-3. However, role of SOCS-3 or PTP-1B in resistance to peripheral effects of leptin remains unclear.

Recently, we examined if prolonged hyperleptinemia may induce resistance to natriuretic effect of leptin in the absence of obesity. Preliminary results suggest that chronic (7-day) administration of exogenous leptin impairs acute increase in natriuresis and decrease in renal medullary Na^+^,K^+^-ATPase activity after subsequent bolus intravenous leptin administration. In addition, pretreatment with α_1_-adrenergic antagonist, prazosin, or NADPH oxidase inhibitor and antioxidant, apocynin, failed to improve these effects, suggesting that neither oxidative stress nor sympathetic nervous system are involved in renal leptin resistance in this model (Bełtowski *et al*. manuscript submitted for publication). In addition, because rats chronically pretreated with leptin have normal or even improved insulin sensitivity, these data suggest that hyperinsulinemia and insulin resistance are not necessary to induce renal leptin resistance in obese animals. This conclusion is in contrast to that obtained by studying the mechanism of resistance to natriuretic effect of dopamine, which is mediated by both hyperinsulinemia and oxidative stress [53, 54].

## ANTINATRIURETIC EFFECT OF CHRONIC HYPERLEPTINEMIA

Although resistance to natriuretic effect of leptin may contribute to the development of hypertension due to unopposed stimulation of the SNS, the effect of chronically elevated leptin on renal function seems more important if pathogenesis of hypertension associated with the metabolic syndrome is considered. Early studies have demonstrated that chronic central or peripheral leptin infusion had no effect on sodium excretion despite elevating blood pressure [6, 55]. These data indicate that leptin impairs pressure-natriuresis relationship - an abnormality common for many types of hypertension.

We examined the effect of leptin administered for 7 days at a dose of 0.5 mg/kg/day (plasma leptin concentration about four times higher than normally) on renal Na^+^ handling in the rat. We found that leptin treatment reduced both absolute and fractional Na^+^ excretion, which was accompanied by the increase in renal cortical and, especially, medullary Na^+^,K^+^-ATPase activity [56, 57]. Further studies have demonstrated that this antinatriuretic effect of leptin is mediated by reactive oxygen species (ROS)-dependent scavenging of intrarenally produced NO. This conclusion is supported by the following observations: (1) leptin increased markers of intrarenal oxidative stress and nitrotyrosine (the product of protein nitration by peroxynitrite, which originates in the reaction between NO and superoxide) concentration, (2) leptin reduced renal NO and its second messenger, cGMP, levels (3) antinatriuretic effect of leptin was abolished by apocynin (NADPH oxidase inhibitor) and tempol (scavenger of superoxide anion radical) [58, 59]. Previously, we [38] and others [60] have demonstrated that NO produced locally within the kidney exerts a tonic inhibitory effect on tubular Na^+^ transport and Na^+^,K^+^-ATPase, and that this effect of NO is limited by superoxide anion radical (O_2_^-•^) continuously produced by NADPH oxidase – a multimeric enzyme which is a predominant source of ROS in the cardiovascular system and the kidney [61, 62]. In addition, α-adrenergic receptor antagonist, prazosion, normalized Na^+^,K^+^-ATPase activity in the renal cortex of leptin-treated rats, indicating that leptin may stimulate sodium pump in part by activating SNS.

## ROLE OF ERKs IN ANTINATRIURETIC EFFECT OF LEPTIN

`To get more insight into the mechanism through which leptin stimulates renal NA^+^,K^+^-ATPase, we examined the effect of leptin infused locally into the renal artery [63]. As mentioned previously [36], short-term (30 min) hormone infusion resulted in the decrease in sodium pump activity specifically in the renal medulla. However, when leptin infusion was prolonged to 3 hours, Na^+^,K^+^-ATPase activity increased in both renal cortex and medulla, indicating that the effect of leptin is time-dependent. Both inhibitory and stimulatory effects of leptin were abolished by JAK inhibitor, AG490. However, only inhibitory effect was abolished by PI3K inhibitors. Unexpectedly, although NADPH oxidase inhibitor, apocynin, abolished the stimulatory effect of leptin, superoxide scavengers, tempol, or superoxide dismutase, augmented it. These results suggest that ROS other than superoxide are involved in Na^+^,K^+^-ATPase stimulation. Because both tempol and SOD convert superoxide to hydrogen peroxide (H2O2), we hypothesized that H2O2 might be involved. Indeed, leptin increased urinary H2O2 excretion, and its stimulatory effect on Na^+^,K^+^-ATPase was abolished by catalase and mimicked by H2O2 [63]. Furthermore, we demonstrated that the effect of either leptin or H2O2 was abolished by extracellular signal-regulated kinase (ERK) inhibitors, PD98059 or U0126, but not by p38 MAPK inhibitor, SB203580. Taken together, these results suggest that the stimulatory effect of locally infused leptin on renal sodium pump is mediated by H2O2-dependent activation of ERK rather than by O_2_^-•^-induced scavenging of NO [63].

## TIME-DEPENDENCY OF THE MECHANISM THROUGH WHICH LEPTIN STIMULATES RENAL Na^+^,K^+^-ATPASE - ROLE OF ANTIOXIDANT ENZYMES

To resolve why O_2_^-•^-NO mechanism predominates after 7-day systemic leptin administration [57] and H_2_O_2_-ERK dependent one after local 3-hour hormone infusion [63], we examined the effect of locally infused leptin in control animals as well as in rats made hyperleptinemic by preceding 7-day systemic leptin treatment [64]. Using combined biochemical and pharmacological approaches, we demonstrated that leptin infused locally to the renal artery stimulated cortical and medullary Na^+^,K^+^-ATPase through the H_2_O_2_-ERK dependent mechanism in normal rats but through the O_2_^-•^-NO mechanism in previously hyperleptinemic rats, which suggests that the mechanism of antinatriuretic effect of leptin changes time-dependently when hormone is administered for prolonged time period. Indeed, in the recent study [65] we found that if leptin was administered systemically, H_2_O_2_-ERK mechanism predominates after 4 days, and O_2_^-•^-NO mechanism after 8 days of hormone administration. The effect of leptin was abolished by NADPH oxidase inhibitor at both time points, however, tempol was effective only if leptin was administered for 8 days but further aggravated its effect after 4 days. In contrast, ERK inhibitor, PD98059, reduced Na^+^,K^+^-ATPase activity and normalized blood pressure in rats receiving leptin for 4 days but not for 8 days These data suggest that leptin induces NADPH oxidase-dependent ROS formation, however, H_2_O_2_-triggered activation of ERK and O_2_^-•^-induced NO deficiency predominate after 4 and 8 days of leptin administration, respectively.

Because NADPH oxidase generates only superoxide which is further converted to H_2_O_2_ by superoxide dismutase (SOD), we looked at the activities of antioxidant enzymes in the kidney of leptin-treated rats. We found that SOD activity was reduced whereas glutathione peroxidase (GPx) activity was increased in the kidney of rats treated with leptin for 8 days but these activities were unchanged in animals receiving leptin for 4 days. This suggests that relative contribution of H_2_O_2_-ERK vs. O_2_^-•^-NO mechanisms is determined by antioxidant enzyme activities. In animals receiving leptin for 4 days, SOD and GPx activities are unchanged which probably makes H_2_O_2_ (more stable than superoxide) the predominant ROS. In contrast, reduced SOD and increased GPx activity after 8 days of leptin administration favors formation of O_2_^-•^ instead of H_2_O_2_ (which is formed by SOD and decomposed by GPx) [65]. Changes of SOD and GPx expression/activities may be a compensatory mechanism aimed to limit oxidative injury since H_2_O_2_ is more damaging than superoxide. Indeed, markers of lipid peroxidation in renal tissue were lower in the 8-day than in the 4-day leptin-treated group [65].

Glutathione (GSH) is a major intracellular antioxidant and ROS sensor. Indeed, the ratio between reduced (GSH) and oxidized (GSSG) glutathione determines oxidation state of protein sulfhydryl groups involved in redox signaling. Therefore, in subsequent studies we asked if glutathione redox status is affected by leptin treatment and how does it contribute to alterations of antioxidant enzymes. In a very recent study [66] we have demonstrated that GSH/GSSG ratio markedly decreases in the kidney of rats receiving leptin for 4 days, whereas in those receiving hormone for 8 days it is partially restored. This restoration is associated with the up-regulation of a rate-limiting enzyme in GSH synthesis, γ-glutamylcysteine synthetase (γ-GCS). Moreover, if GSH synthesis is suppressed by γ-GCS inhibitor, buthionine sulfoximine, leptin-induced changes of SOD and GPx are accelerated, i.e. occur already after 4 days of hormone administration, which is accompanied by the shift from H_2_O_2_-ERK to O_2_^-•^-NO dependent mechanism of leptin-induced antinatriuresis and Na^+^,K^+^-ATPase stimulation. In contrast, if GSH precursor, N-acetylcysteine, is administered together with leptin, it partially corrects fall in GSH/GSSG ratio and prevents changes in SOD and GPx even at the 8^th^ day of leptin treatment. Consequently, H_2_O_2_-ERK dependent mechanism still operates in the 8-day leptin group [66]. Taken together, these results indicate that changes in GSH redox status determine decrease in SOD and increase in GPx activities after leptin treatment, which has profound consequences for the mechanism through which leptin reduces natriuresis and elevates blood pressure (Fig. (**1**)).

## EFFECT OF GRADUAL INCREASE IN PLASMA LEPTIN CONCENTRATION

In all studies mentioned above leptin was administered at a constant dose of 0.5 mg/kg/day. Because in patients with the metabolic syndrome plasma leptin increases progresssively other the long period of time, gradually increasing leptin dosage seems to be a more physiologically relevant approach. We demonstrated that if leptin is administered at progressively increasing doses, SOD and GPx activities are unchanged even after 12 days of hormone administration. Although renal Na^+^,K^+^-ATPase activity and blood pressure are elevated to the similar extent as in rats receiving leptin at a constant dose, H_2_O_2_-ERK mechanism still operates after 12 days of leptin treatment at progressively increasing doses. It is likely that if leptin dose is progressively increased, the cells can better handle the oxidative stress, e.g. by nonenzymatic antioxidants, and compensatory changes of SOD and GPx do not occur [66].

These considerations are potentially important from therapeutic point of view. Although oxidative stress is clearly involved in the pathogenesis of cardiovascular diseases, the clinical outcome in trials with antioxidants was generally disappointing [67]. One of the reasons may be that some antioxidants such as tempol scavenge only some specific ROS and may even generate the others. It seems that more selective antioxidant therapy, targeting specific ROS and signaling pathways triggered by them, may be a more appropriate therapeutic approach than nonspecific antioxidants such as ascorbate or vitamin E examined in previous trials.

## ROLE OF EPIDERMAL GROWTH FACTOR RECEPTOR (EGFR) IN RENAL EFFECT OF LEPTIN

Many mechanisms of H_2_O_2_-induced activation of ERKs have been described in the literature, and ERKs have been implicated in the pathogenesis of various forms of experimental hypertension. To unravel the mechanism through which leptin activates ERKs in the kidney, we focused on epidermal growth factor receptor (EGFR). EGFR is a member of membrane-bound receptor tyrosine kinase family which is abundantly expressed in the vascular wall and renal tubular cells. Upon ligand binding, EGFR undergoes autophosphorylation at several tyrosine residues within its intracellular domain and triggers various signaling pathways including ERK. Stimulation of EGFR results in the recruitment of adaptor proteins Grb2 and Sos, which interact with and activate membrane-bound Ras protein. Activated Ras stimulates phosphorylation cascade including protein kinases Raf, MEK and ERK1/2 [68]. Several lines of evidence suggest the role of EGFR in arterial hypertension. First, apart from its cognate ligand, EGFR may be activated by many mediators involved in the regulation of vascular tone and renal Na^+^ transport such as angiotensin II, endothelin-1 and norepinephrine; the phenomenon referred to as transactivation. Second, increased expression and/or activity of EGFR were reported in various experimental models of hypertension, and EGFR inhibitors reduce blood pressure in some of these models [69]. Finally, leptin has been demonstrated to transactivate EGFR in several cancer cell lines as well as in vascular smooth muscle cells [70].

We have demonstrated that the effect of leptin on renal ERK and Na^+^,K^+^-ATPase is abolished by EGFR inhibitors, AG1478 or PD158780, and is mimicked by EGF [71]. There are two major mechanisms of EGFR transactivation: (1) stimulation of metalloproteases which generate peptide EGFR ligands from their membrane-bound precursors, and (2) activation by reactive oxygen species of non-receptor tyrosine kinase Src, which phosphorylates intracellular domain of EGFR. Therefore, we further studied which of these mechanisms is triggered by leptin. We found that leptin stimulates EGFR phosphorylation at Tyr^845^ (the principal Src phosphorylation site) and, to a lesser extent, at Tyr^1068^ (one of autophosphorylation sites) [72]. In addition, the effects of leptin on EGFR phosphorylation, ERK phosphorylation and renal Na^+^,K^+^-ATPase were abolished by specific Src inhibitor, PP2, as well as by H_2_O_2_ scavenger, catalase, but not by metalloprotease inhibitor, GM6001. Both leptin and H_2_O_2_ increased Src phosphorylation at Tyr^418^ (a marker of Src activity). Taken together, these results indicate that leptin-induced activation of EGFR is mediated by ROS (presumably H_2_O_2_), and Src kinase [71, 72].

Finally, we asked if inhibitors of EGFR-ERK pathway can normalize renal Na^+^,K^+^-ATPase activity, natriuresis and blood pressure in rats receiving leptin. For this purpose, leptin was administered at progressively increasing doses for 10 days and blood pressure and renal Na^+^,K^+^-ATPase were measured. We found that apocynin, PP2 or AG1478 reduced blood pressure, increased Na^+^ excretion and decreased renal Na^+^,K^+^-ATPase activity in leptin-treated animals. In contrast, GM6001 did not attenuate leptin-induced increase in EGFR phosphorylation nor did it modulate the effect of leptin on renal sodium pump or sodium handling. These data indicate that inhibiting Src-EGFR pathway may be a potential therapeutic strategy for arterial hypertension associated with hyperleptinemic states such as metabolic syndrome or type 2 diabetes.

## RENAL RESISTANCE TO NATRIURETIC PEPTIDES IN CHRONIC HYPERLEPTINEMIA

Apart from NO, atrial natriuretic peptide (ANP) and a related intrarenally produced peptide, urodilatin, inhibit Na^+^ reabsorption through the cGMP-PKG dependent mechanism [73]. Interestingly, in our model of chronic hyperleptinemia plasma ANP and urinary urodilatin concentrations were increased [56, 57]. Reduced natriuresis and renal cGMP generation despite increased ANP suggest that the kidneys become resistant to natriuretic peptides following leptin treatment. Indeed, increase in cGMP and Na^+^ excretion in response to exogenous ANP was less marked in hyperleptinemic than in control rats [74]. This effect was corrected by inhibitor of cGMP-specific phosphodiesterase (PDE5), zaprinast, suggesting that enhanced cGMP degradation by PDE5 is responsible for this phenomenon. In addition, resistance to ANP and its correction by zaprinast were observed in rats made obese by high-calorie diet thus indicating that hyperleptinemia could contribute to renal unresponsiveness to natriuretic peptides observed in obese subjects [74]. Although increased PDE5-dependent cGMP degradation is a common mechanism of renal unresponsiveness to ANP in various disease states, the mechanism through which leptin enhances PDE5 is unclear. Interestingly, natriuretic effect of synthetic NO donor was intact in either leptin-treated or obese rats, indicating that various intracellular pools of cGMP mediate the effects of ANP and NO on renal Na^+^ transport.

## LEPTIN AND THE REGULATION OF RENAL OUABAIN-RESISTANT NA^+^-ATPASE IN PHYSIOLOGICAL AND PATHOLOGICAL CONDITIONS

Apart from Na^+^,K^+^-ATPase, renal tubules contain the second sodium pump, ouabain-resistant Na^+^-ATPase. Unlike Na^+^,K^+^-ATPase, Na^+^-ATPase transports sodium but not potassium, in not inhibited by Na^+^,K^+^-ATPase inhibitor, ouabain, but is sensitive to loop diuretics such as furosemide, and is expressed only in the proximal tubule but not in other nephron segments [75]. Although this enzyme transport only 10-15% of sodium reabsorbed in the proximal tubule, changes in its activity may cause marked alterations of natriuresis. The mechanisms regulating Na^+^-ATPase are poorly characterized; however, its activity is affected by many mediators controlling Na^+^ balance such as angiotensin II, nitric oxide and natriuretic peptides [75].

We have demonstrated that acutely administered leptin does not change renal Na^+^-ATPase activity suggesting that this pump is not involved in acute natriuretic effect of leptin [76]. In contrast, Na^+^-ATPase activity was increased in the renal cortex of rats treated with leptin for 7 days [76]. Using pharmacological inhibitors of various signaling pathways, we have demonstrated that, similarly to Na^+^,K^+^-ATPase, Na^+^-ATPase stimulation in hyperleptinemic rats results from O_2_^-^ -dependent NO deficiency. However, in contrast to Na^+^,K^+^-ATPase which is inhibited by NO in the cGMP-PKG dependent manner, Na^+^-ATPase is inhibited by NO-cGMP pathway through the PKG-independent mechanism. Rather, cGMP stimulates phosphodiesterase 2 which degrades cAMP and removes its tonic stimulatory effect on ouabain-resistant Na^+^ pump [77]. Leptin stimulates ROS formation leading to NO degradation, and thus downregulates this inhibitory mechanism. Consequently, the stimulatory effect of cAMP on ouabain-resistant Na^+^-ATPase is enhanced (Fig. (**2**)).

## CLINICAL AND THERAPEUTIC IMPLICATIONS

Although the relationship between high serum leptin and hypertension has been reported in many human studies [78, 79], it is unknown if chronic hyperleptinemia affects renal function in humans. In one study [80], plasma leptin was inversely correlated with lithium clearance, which suggests that high leptin was associated with increased Na^+^ reabsorption in the proximal tubule. These findings suggest that chronically elevated leptin has antinatriuretic effect in humans.

Given all data presented above, it could be suggested that two therapeutic strategies may be proposed to target leptin signaling in hypertension: (1) improving beneficial natriuretic effect of this hormone, i.e. reducing renal leptin resistance, (2) reducing chronically elevated leptin in patients with the metabolic syndrome or interrupting leptin signaling involved in the enhancement of Na^+^ reabsorption to reduce detrimental effect of chronic hyperleptinemia. Until now, only weight loss seems to fulfill both these criteria. Currently it is unclear how we could improve natriuretic effect of leptin. Possibly, targeting intracellular pathways involved in leptin resistance such as SOCS-3 or PTP1B may be a useful strategy in the future. More promising seems to be inhibiting signaling pathways triggered by chronic hyperleptinemia which drive enhanced Na^+^ reabsorption, e.g. by EGFR, ERK or Src inhibitors, as well as by antioxidants.

However, leptin signaling may also be affected by less specific therapies. The effect of many currently used antihypertensive drugs on plasma leptin has been examined. However, interpretation of results is not easy since decrease in plasma leptin can result from various mechanisms such as: (1) direct inhibition of leptin production by adipose tissue, (2) compensatory response of adipose tissue to improved leptin signaling in the hypothalamus, (3) an indirect effect of the drug resulting for example from changes in adiposity, insulin sensitivity, etc. Until now, it has been demonstrated that at least some angiotensin-converting enzyme inhibitors, angiotensin AT_1_ receptor antagonists, calcium antagonists and α-adrenergic receptor antagonists decrease plasma leptin level [81]. The effect of β-adrenergic receptor antagonists is controversial since both increase and decrease in leptin have been reported, whereas thiazide diuretics have no effect. It is possible that modulation of leptin signaling contributes to beneficial effect of some of these therapies on the cardiovascular system.

## Figures and Tables

**Fig. (1):**
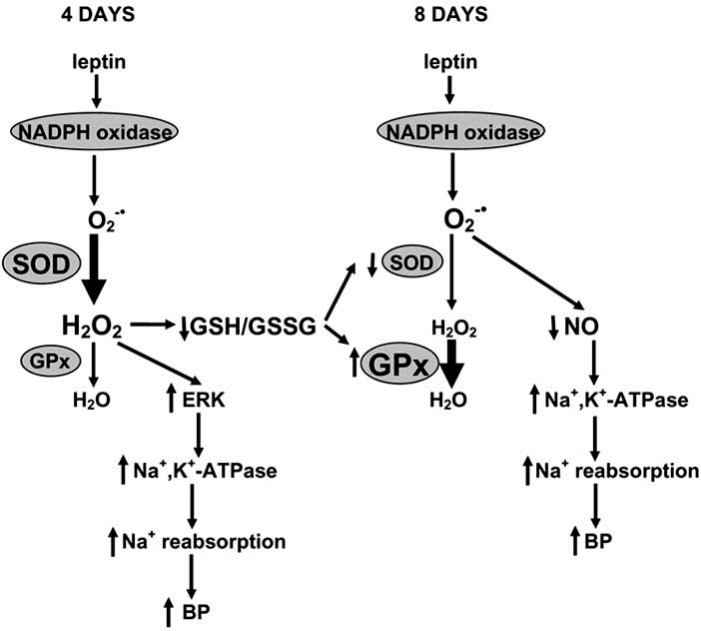
The mechanism of leptin-induced stimulation of renal Na^+^,K^+^-ATPase and BP elevation in rats receiving a constant dose of leptin for 4 or 8 days. Leptin stimulates superoxide (O_2_^-•^) production by NADPH oxidase. In the 4-day group, O_2_^-•^ is rapidly converted by superoxide dismutase (SOD) to hydrogen peroxide (H_2_O_2_), which stimulates extracellular signal-regulated kinases (ERK). ERK activate renal Na^+^,K^+^-ATPase leading to the increase in Na^+^ reabsorption and BP elevation. Simultaneously, H_2_O_2_ and/or H_2_O_2_-induced oxidative stress reduces the ratio between reduced glutathione (GSH) and glutathione disulfide (GSSG). Reduced GSH/GSSG ratio induces decrease in SOD and increase in GPx activity. Consequently, in the 8-day group H_2_O_2_ is efficiently metabolized while O_2_^-•^ becomes a predominant ROS formed. Superoxide reacts with nitric oxide (NO) to form peroxynitrite (ONOO^-^). Because NO inhibits renal Na^+^,K^+^-ATPase, its deficiency results in Na^+^,K^+^-ATPase stimulation and BP elevation.

**Fig. (2):**
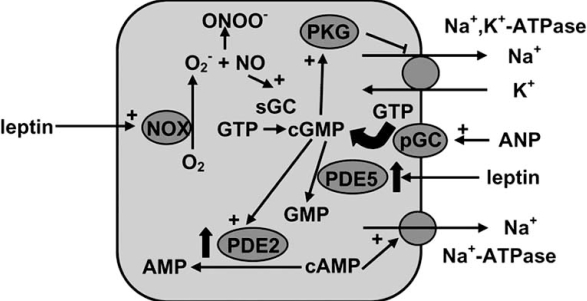
Effect of chronic hyperleptinemia on renal tubular Na^+^-transporting pumps. Leptin stimulates NADPH oxidase (NOX) which generates superoxide anion radical (O_2_^-^). Superoxide reacts with nitric oxide (NO) to form peroxynitrite (ONOO^-^) and thus curtails the inhibitory effect of NO on active sodium reabsorption. NO stimulates soluble guanylyl cyclase (sGC) to generate cGMP, and cGMP inhibits both Na^+^,K^+^-ATPase and ouabain-resistant Na^+^-ATPase. However, Na^+^,K^+^-ATPase is inhibited in protein kinase G (PKG)-dependent manner whereas Na^+^-ATPase is inhibited because cGMP stimulates (in PKG-independent manner) phosphodiesterase 2 (PDE2) and decreases cAMP concentration. Apart from NO, cGMP synthesis is stimulated by atrial natriuretic peptide (ANP) binding to membrane receptors (particulate guanylyl cyclase, pGC). Hyperleptinemia induces resistance to ANP by stimulating degradation of cGMP by phosphodiesterase 5 (PDE5). The scheme presents mechanisms operating in different nephron segments; Na^+^,K^+^-ATPase is inhibited by ANP in the medullary collecting duct whereas Na^+^-ATPase is expressed only in the proximal tubule. Localization of ANP and leptin receptors demonstrated above does not necessarily reproduce their real localization in polarized tubular cells but was chosen arbitrarily for better clarity.

## References

[1] Genovesi S, Pieruzzi F (2006). Obesity-associated hypertension in childhood: A new epidemic problem. Curr Hypertens Rev.

[2] Seki G, Yamada H, Li Y, Horita S, Suzuki M, Toshiro F (2008). The roles of abnormal renal sodium handling in hypertension associated with metabolic syndrome. Curr Hypertens Rev.

[3] Wilson AM, Ryan MC (2007). The role of adipokines in hypertension and cardiovascular disease. Curr Hypertens Rev.

[4] Zhang Y, Proenca R, Maffei M, Barone M, Leopold L, Friedman JM (1994). Positional cloning of the mouse obese gene and its human homologue. Nature.

[5] Myers MG, Cowley MA, Münzberg H (2008). Mechanisms of leptin action and leptin resistance. Annu Rev Physiol.

[6] Shek EW, Brands MW, Hall JE (1998). Chronic leptin infusion increases arterial pressure. Hypertension.

[7] Aizawa-Abe M, Ogawa Y, Masuzaki H (2000). Pathophysiological role of leptin in obesity-related hypertension. J Clin Invest.

[8] Mark AL, Shaffer RA, Correia ML, Morgan DA, Sigmund CD, Haynes WG (1999). Contrasting blood pressure effects of obesity in leptin-deficient ob/ob mice and agouti yellow obese mice. J Hypertens.

[9] Agata J, Masuda A, Takada M (1997). High plasma immunoreactive leptin level in essential hypertension. Am J Hypertens.

[10] Meneton P, Jeunemaitre X, de Wardener HE, MacGregor GA (2005). Links between dietary salt intake, renal salt handling, blood pressure, and cardiovascular diseases. Physiol Rev.

[11] Féraille E, Doucet A (2001). Sodium-potassium-adenosinetriphosphatase-dependent sodium transport in the kidney: hormonal control. Physiol Rev.

[12] Garg LC, Narang N, McArdle S (1985). Na-K-ATPase in nephron segments of rats developing spontaneous hypertension. Am J Physiol.

[13] Beach RE, DuBose TJ (1990). Adrenergic regulation of Na^+^,K^+^-ATPase activity in proximal tubules of spontaneously hypertensive rats. Kidney Int.

[14] Magyar CE, Zhang Y, Holstein-Rathlou NH, McDonough AA (2000). Proximal tubule Na transporter responses are the same during acute and chronic hypertension. Am J Physiol Renal Physiol.

[15] Hayward AL, Hinojos CA, Nurowska B (1999). Altered sodium pump alpha and gamma subunit gene expression in nephron segments from hypertensive rats. J Hypertens.

[16] Hinojos CA, Doris PA (2004). Altered subcellular distribution of Na^+^,K^+^-ATPase in proximal tubules in young spontaneously hypertensive rats. Hypertension.

[17] Ruiz-Opazo N, Barany F, Hirayama K, Herrera VL (1994). Confirmation of mutant alpha 1 Na,K-ATPase gene and transcript in Dahl salt-sensitive/JR rats. Hypertension.

[18] Orosz DE, Hopfer U (1996). Pathophysiological consequences of changes in the coupling ratio of Na,K-ATPase for renal sodium reabsorption and its implications for hypertension. Hypertension.

[19] Kaneko Y, Cloix JF, Herrera VL, Ruiz-Opazo N (2005). Corroboration of Dahl S Q276L alpha1Na,K-ATPase protein sequence: impact on affinities for ligands and on E1 conformation. J Hypertens.

[20] Nishi A, Eklöf AC, Bertorello AM, Aperia A (1993). Dopamine regulation of renal Na^+^,K^+^-ATPase activity is lacking in Dahl salt-sensitive rats. Hypertension.

[21] Hussain T, Kansra V, Lokhandwala MF (1999). Renal dopamine receptor signaling mechanisms in spontaneously hypertensive and Fischer 344 old rats. Clin Exp Hypertens.

[22] Manunta P, Citterio L, Lanzani C, Ferrandi M (2007). Adducin polymorphisms and the treatment of hypertension. Pharmacogenomics.

[23] Ferrandi M, Salardi S, Tripodi G (1999). Evidence for an interaction between adducin and Na^+^-K^+^-ATPase: relation to genetic hypertension. Am J Physiol.

[24] Efendiev R, Krmar RT, Ogimoto G (2004). Hypertension-linked mutation in the adducin alpha-subunit leads to higher AP2-mu2 phosphorylation and impaired Na^+^,K^+^-ATPase trafficking in response to GPCR signals and intracellular sodium. Circ Res.

[25] Torielli L, Tivodar S, Montella RC (2008). alpha-Adducin mutations increase Na/K pump activity in renal cells by affecting constitutive endocytosis: implications for tubular Na reabsorption. Am J Physiol Renal Physiol.

[26] Marwaha A, Lokhandwala MF (2003). Diminished natriuretic response to dopamine D1 receptor agonist, SKF-38393 in obese Zucker rats. Clin Exp Hypertens.

[27] Shah S, Hussain T (2006). Enhanced angiotensin II-induced activation of Na^+^,K^+^-ATPase in the proximal tubules of obese Zucker rats. Clin Exp Hypertens.

[28] Strazzullo P, Barba G, Cappuccio FP (2001). Altered renal sodium handling in men with abdominal adiposity: a link to hypertension. J Hypertens.

[29] Dessì-Fulgheri P, Sarzani R, Serenelli M (1999). Low calorie diet enhances renal, hemodynamic, and humoral effects of exogenous atrial natriuretic peptide in obese hypertensives. Hypertension.

[30] Serradeil-Le Gal C, Raufaste D, Brossard G (1997). Characterization and localization of leptin receptors in the rat kidney. FEBS Lett.

[31] Bełtowski J, Wójcicka G, Górny D, Marciniak A (2002). Human leptin administered intraperitoneally stimulates natriuresis and decreases renal medullary Na^+^, K^+^-ATPase activity in the rat -- impaired effect in dietary-induced obesity. Med Sci Monit.

[32] Villarreal D, Reams G, Freeman RH, Taraben A (1998). Renal effects of leptin in normotensive, hypertensive, and obese rats. Am J Physiol.

[33] Bełtowski J, Jochem J, Wójcicka G, Żwirska-Korczala K (2004). Influence of intravenously administered leptin on nitric oxide production, renal hemodynamics and renal function in the rat. Regul Pept.

[34] Jackson EK, Li P (1997). Human leptin has natriuretic activity in the rat. Am J Physiol.

[35] Bełtowski J, Wójcicka G (2002). Spectrophotometric method for the determination of renal ouabain-sensitive H^+^,K^+^-ATPase activity. Acta Biochim Pol.

[36] Bełtowski J, Marciniak A, Wójcicka G (2004). Leptin decreases renal medullary Na^+^, K^+^-ATPase activity through phosphatidylinositol 3-kinase dependent mechanism. J Physiol Pharmacol.

[37] Martinez-Anso E, Lostao MP, Martinez JA (1999). Immunohistochemical localization of leptin in rat kidney. Kidney Int.

[38] Bełtowski J, Marciniak A, Wójcicka G, Górny D (2003). Nitric oxide decreases renal medullary Na^+^,K^+^-ATPase activity through cyclic GMP-protein kinase G dependent mechanism. J Physiol Pharmacol.

[39] Varela M, Herrera M, Garvin JL (2004). Inhibition of Na-K-ATPase in thick ascending limbs by NO depends on O2- and is diminished by a high-salt diet. Am J Physiol Renal Physiol.

[40] Hakam AC, Hussain T (2006). Angiotensin II AT_2_ receptors inhibit proximal tubular Na^+^-K^+^-ATPase activity *via* a NO/cGMP-dependent pathway. Am J Physiol Renal Physiol.

[41] Bełtowski J, Wójcicka G, Jamroz-Wiśniewska A, Borkowska E (2007). Role of PI3K and PKB/Akt in acute natriuretic and NO-mimetic effects of leptin. Regul Pept.

[42] Chibalin AV, Zierath JR, Katz AI, Berggren PO, Bertorello AM (1998). Phosphatidylinositol 3-kinase-mediated endocytosis of renal Na^+^,K^+^-ATPase alpha subunit in response to dopamine. Mol Biol Cell.

[43] Yudowski GA, Efendiev R, Pedemonte CH, Katz AI, Berggren PO, Bertorello AM (2000). Phosphoinositide-3 kinase binds to a proline-rich motif in the Na^+^,K^+^-ATPase alpha subunit and regulates its trafficking. Proc Natl Acad Sci USA.

[44] Efendiev R, Chen Z, Krmar RT (2005). The 14-3-3 protein translates the Na^+^,K^+^-ATPase α1-subunit phosphorylation signal into binding and activation of phosphoinositide 3-kinase during endocytosis. J Biol Chem.

[45] Villarreal D, Reams G, Freeman R (2006). Leptin blockade attenuates sodium excretion in saline-loaded normotensive rats. Mol Cell Biochem.

[46] Dobrian AD, Schriver SD, Lynch T, Prewitt RL (2003). Effect of salt on hypertension and oxidative stress in a rat model of diet-induced obesity. Am J Physiol Renal Physiol.

[47] Correia ML, Haynes WG, Rahmouni K, Morgan DA, Sivitz WI, Mark AL (2002). The concept of selective leptin resistance: evidence from agouti yellow obese mice. Diabetes.

[48] Rahmouni K, Haynes WG, Morgan DA, Mark AL (2002). Selective resistance to central neural administration of leptin in agouti obese mice. Hypertension.

[49] Rahmouni K, Morgan DA, Morgan GM, Mark AL, Haynes WG (2005). Role of selective leptin resistance in diet-induced obesity hypertension. Diabetes.

[50] Coatmellec-Taglioni G, Dausse JP, Giudicelli Y, Ribiere C (2003). Sexual dimorphism in cafeteria diet-induced hypertension is associated with gender-related difference in renal leptin receptor down-regulation. J Pharmacol Exp Ther.

[51] Gu JW, Wang J, Stockton A, Lokitz B, Henegar L, Hall JE (2004). Cytokine gene expression profiles in kidney medulla and cortex of obese hypertensive dogs. Kidney Int.

[52] Villarreal D, Reams G, Freeman RH (2000). Effects of renal denervation on the sodium excretory actions of leptin in hypertensive rats. Kidney Int.

[53] Banday AA, Marwaha A, Tallam LS, Lokhandwala MF (2005). Tempol reduces oxidative stress, improves insulin sensitivity, decreases renal dopamine D1 receptor hyperphosphory­lation, and restores D1 receptor-G-protein coupling and function in obese Zucker rats. Diabetes.

[54] Umrani DN, Banday AA, Hussain T, Lokhandwala MF (2002). Rosiglitazone treatment restores renal dopamine receptor function in obese Zucker rats. Hypertension.

[55] Kuo JJ, Jones OB, Hall JE (2001). Inhibition of NO synthesis enhances chronic cardiovascular and renal actions of leptin. Hypertension.

[56] Bełtowski J, Jamroz-Wiśniewska A, Borkowska E, Wójcicka G (2004). Up-regulation of renal Na^+^,K^+^-ATPase: the possible novel mechanism of leptin-induced hypertension. Pol J Pharmacol.

[57] Beltowski J, Wójcicka G, Marciniak A, Jamroz A (2004). Oxidative stress, nitric oxide production, and renal sodium handling in leptin-induced hypertension. Life Sci.

[58] Bełtowski J, Jamroz-Wiśniewska A, Borkowska E, Nazar J, Marciniak A (2005). Antioxidant treatment normalizes renal Na^+^,K^+^-ATPase activity in leptin-treated rats. Pharmacol Rep.

[59] Beltowski J, Wójcicka G, Jamroz-Wiśniewska A, Borkowska E, Marciniak A (2005). Antioxidant treatment normalizes nitric oxide production, renal sodium handling and blood pressure in experimental hyperleptinemia. Life Sci.

[60] Herrera M, Ortiz PA, Garvin JL (2006). Regulation of thick ascending limb transport: role of nitric oxide. Am J Physiol Renal Physiol.

[61] Garvin JL, Ortiz PA (2003). The role of reactive oxygen species in the regulation of tubular function. Acta Physiol Scand.

[62] Bełtowski J, Marciniak A, Jamroz-Wiśniewska A, Borkowska E (2004). Nitric oxide -- superoxide cooperation in the regulation of renal Na^+^,K^+^-ATPase. Acta Biochim Pol.

[63] Marciniak A, Jamroz-Wiśniewska A, Borkowska E, Bełtowski J (2005). Time-dependent effect of leptin on renal Na^+^,K^+^-ATPase activity. Acta Biochim Pol.

[64] Marciniak A, Borkowska E, Kedra A, Rychlik M, Beltowski J (2006). Time-dependent transition from H_2_O_2_-extracellular signal-regulated kinase- to O­-nitric oxide-dependent mechanisms in the stimulatory effect of leptin on renal Na^+^K^+^-ATPase in the rat. Clin Exp Pharmacol Physiol.

[65] Wójcicka G, Jamroz-Wiśniewska A, Widomska S, Ksiazek M, Bełtowski J (2008). Role of extracellular signal-regulated kinases (ERK) in leptin-induced hypertension. Life Sci.

[66] Bełtowski J, Jamroz-Wiśniewska A, Wójcicka G, Lowicka E, Wojtak A (2008). Renal antioxidant enzymes and glutathione redox status in leptin-induced hypertension. Mol Cell Biochem.

[67] Meydani M (2004). Vitamin E modulation of cardiovascular disease. Ann N Y Acad Sci.

[68] Fernandez-Patron C (2007). Therapeutic potential of the epidermal growth factor receptor transactivation in hypertension: a convergent signaling pathway of vascular tone, oxidative stress, and hypertrophic growth downstream of vasoactive G-protein-coupled receptors?. Can J Physiol Pharmacol.

[69] Kim J, Lee CK, Park HJ (2006). Epidermal growth factor induces vasoconstriction through the phosphatidylinositol 3-kinase-mediated mitogen-activated protein kinase pathway in hypertensive rats. J Pharmacol Sci.

[70] Chao HH, Hong HJ, Liu JC (2007). Leptin stimulates endothelin-1 expression *via* extracellular signal-regulated kinase by epidermal growth factor receptor transactivation in rat aortic smooth muscle cells. Eur J Pharmacol.

[71] Bełtowski J, Wójcicka G, Trzeciak J, Marciniak A (2006). H_2_O_2_ and Src-dependent transactivation of the EGF receptor mediates the stimulatory effect of leptin on renal ERK and Na^+^,K^+^-ATPase. Peptides.

[72] Jamroz-Wiśniewska A, Wójcicka G, Łowicka E, Książek M, Bełtowski J (2008). Transactivation of epidermal growth factor receptor in vascular and renal systems in rats with experimental hyperleptinemia: role in leptin-induced hypertension. Biochem Pharmacol.

[73] Bełtowski J, Wójcicka G (2002). Regulation of renal tubular sodium transport by cardiac natriuretic peptides: two decades of research. Med Sci Monit.

[74] Bełtowski J, Jamroz-Wisniewska A, Borkowska E, Marciniak A (2006). Phosphodiesterase 5 inhibitor ameliorates renal resistance to atrial natriuretic peptide associated with obesity and hyperleptinemia. Arch Med Res.

[75] De Souza AM, Carvalho TL, Sabino PM (2007). Characterization and partial isolation of ouabain-insensitive Na^+^-ATPase in MDCK I cells. Biochimie.

[76] Bełtowski J, Jamroz-Wiśniewska A, Nazar J, Wójcicka G (2004). Spectrophotometric assay of renal ouabain-resistant Na^+^-ATPase and its regulation by leptin and dietary-induced obesity. Acta Biochim Pol.

[77] Bełtowski J, Borkowska E, Wójcicka G, Marciniak A (2007). Regulation of renal ouabain-resistant Na^+^-ATPase by leptin, nitric oxide, reactive oxygen species, and cyclic nucleotides: implications for obesity-associated hypertension. Clin Exp Hypertens.

[78] Patel SB, Reams GP, Spear RM, Freeman RH, Villarreal D (2008). Leptin: linking obesity, the metabolic syndrome, and cardiovascular disease. Curr Hypertens Rep.

[79] Bełtowski J (2006). Role of leptin in blood pressure regulation and arterial hypertension. J Hypertens.

[80] El-Gharbawy AH, Kotchen JM, Grim CE (2002). Gender-specific correlates of leptin with hypertension-related phenotypes in African Americans. Am J Hypertens.

[81] Koh KK, Park SM, Quon MJ (2008). Leptin and cardiovascular disease: response to therapeutic interventions. Circulation.

